# Outbreak of *Ralstonia pickettii* associated with contamination of saline products distributed internationally, the United Kingdom, 2024

**DOI:** 10.2807/1560-7917.ES.2024.29.27.2400384

**Published:** 2024-07-04

**Authors:** Mike Saunders, Amy Weaver, Rebecca Stretch, D Jeyaratnam, Mariyam Mirfenderesky, David Elliott, Charlotte Patterson, David Williams, Dervla TD Kenna, Jack Turton, Karen L Osman, Jane F Turton, Colin S Brown, JWT Elston

**Affiliations:** 1 United Kingdom Health Security Agency (UKHSA), Colindale, United Kingdom; * These authors contributed equally to this work and share first authorship.

**Keywords:** *Ralstonia pickettii*, contamination, healthcare associated infection, sepsis, infection control

## Abstract

We describe an outbreak of *Ralstonia pickettii* in the United Kingdom, with isolates genetically indistinguishable from a 2023 Australian outbreak linked to internationally distributed saline solutions. Confirmed cases (n = 3) had bacteraemia, clinically relevant infection, indwelling venous lines and frequent healthcare contact. Multi-stakeholder intervention was required including product recall and risk communications. We recommend a low threshold for investigating clusters of *Ralstonia* species and similar opportunistic pathogens, considering contaminated product sources. Effective mitigation requires multi-agency partnership and international collaboration.

We describe the detection and investigation of an outbreak of *Ralstonia pickettii* in the United Kingdom (UK) linked to a 2023 outbreak in Australia, associated with internationally distributed saline products. We aim to alert international readers to the possibility of linked cases elsewhere, describe steps taken to investigate and mitigate the UK outbreak and highlight risks of product contamination, key implications and recommendations for public health institutions.

## Outbreak notification

In late 2023, the UK Health Security Agency (UKHSA) was notified by the Australian counterparts of a national outbreak of hospital-acquired *R. pickettii* linked to saline products used for irrigation, inhalation and eyewash, which were supplied also to the UK. Following product testing, the Australian medicines regulator oversaw product recall and implemented a country-specific intervention of additionally suspending products produced by the manufacturer [1,2]. The UKHSA was also made aware of a potentially associated investigation in Germany [3].

## Outbreak investigation

National laboratory surveillance data over the preceding 3 years in England were reviewed to identify cases of *R. pickettii* and *Ralstonia* species, where local laboratories were unable to identify isolates at species level. The surveillance system captures routine data on infectious diseases, including organism identified, specimen information and case demographics, from diagnostic laboratories across England, with nearly complete national coverage. It is a requirement that all clinically relevant isolates from sterile sites are reported. There were no signs of exceedance in *R. pickettii* or *Ralstonia* spp. to signal an underlying outbreak in England at the time of notification. The UKHSA maintained surveillance and actively investigated *Ralstonia* spp. cases.

Relevant and available isolates since 1 April 2021 were requested for submission to the UKHSA Antimicrobial Resistance and Healthcare Associated Infections (AMRHAI) reference laboratory for identification, typing by pulsed-field gel electrophoresis (PFGE) following *Xbal* restriction and comparative analysis of whole genome sequencing (WGS) data. Illumina (Illumina Inc, San Diego, the United States) sequence reads were trimmed using Trimmomatic (version 0.32 http://www.usadellab.org/cms/?page=trimmomatic) and de novo assembled using SPAdes (3.15.5 using the –careful flag https://github.com/ablab/spades). An initial analysis selected genome sequences similar to the Australia isolate (ASM3380453v1; within 0.1 MASH (https://github.com/marbl/Mash distance between assemblies; version 2.3 with sketch size 5,000, *k*-mer size 21) from those publicly available via National Center for Biotechnology Information (NCBI, https://www.ncbi.nlm.nih.gov/) datasets-cli tool (version 16.6.0) with taxon *Ralstonia pickettii*. A core genome alignment was constructed from this selection using parsnp (1.7.4) and pairwise single nucleotide polymorphism (SNP) differences were calculated using snp-dists (0.8.2). The analysis was corroborated for the UK isolates with a read mapping approach against ASM3380453v1 as a reference, using snippy (4.6.0). In the case of isolate (UK confirmed 12/2023 1), which had a lower sequencing yield than the other isolates, the read alignments produced with snippy were used to discount a number of SNPs (n = 15) in the parsnp alignment as likely artefacts. Case definitions were developed (Box) with a hypothesis that confirmed cases had been exposed to the same products produced by the manufacturer associated with the 2023 outbreak in Australia.

Box **Confirmed and probable case definitions**

**Confirmed case:**

• Any person in the United Kingdom from whom a laboratory-confirmed isolate of *Ralstonia pickettii* of the same cluster as the Australian outbreak strain^a^ was retrieved.

**Probable case:**

• Any person in the United Kingdom who had a laboratory-confirmed isolate of *Ralstonia pickettii* where genotyping or further speciation was pending or not completed and with a specimen date from 1 July 2023 onwards^b^.

or

• Any person in the United Kingdom who had a laboratory-confirmed isolate of *Ralstonia* species where genotyping was pending or not completed, with a specimen date from July 2023 onwards and with an epidemiological link to a confirmed case (e.g. same hospital) or known to have been exposed to saline products produced by the manufacturer associated with the 2023 Australia outbreak.

^a^ Defined by whole genome sequencing and bioinformatic analysis.
^b^ Date from which products associated with the 2023 Australia outbreak were known to be contaminated.

We sent targeted questionnaires to affected healthcare settings with a confirmed or probable case to gather exposure histories, focusing on exposures to saline product(s). We exchanged information with international partners to gather intelligence, improve case finding and identify the source of exposure.

## Investigation findings

**Figure 1:**
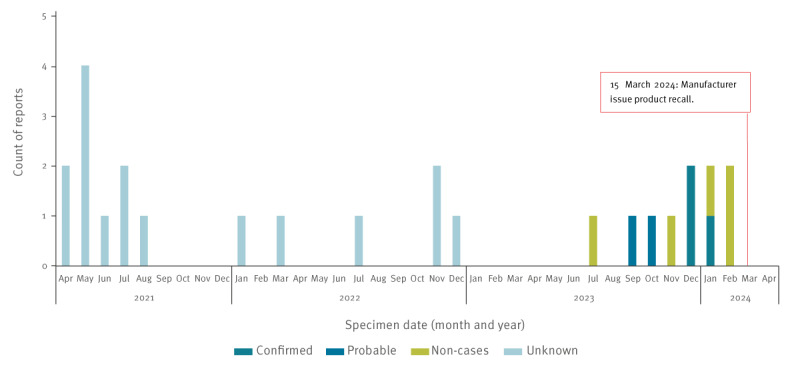
Temporal distribution of *Ralstonia pickettii* reports, United Kingdom, 1 April 2021–1 April 2024 (n = 26)

To date, UKHSA has identified three confirmed and two probable cases with specimen dates 14 September 2023–23 January 2024 (Table and Figure 1). A further five cases that initially met the probable case definition were investigated and ruled out (labelled non-cases in Figure 1).

**Table:** Characteristics of confirmed and probable cases with *Ralstonia pickettii*, United Kingdom, September 2023–January 2024 (n = 5)

Characteristics	Confirmed n = 3	Probable n = 2
Age category (years)		
0–19	0	1
20–39	0	0
40–59	2	0
> 60	1	1
Sex		
Male	1	2
Female	2	0
Specimen type		
Blood	3	0
Swab^a^	0	2
Clinically relevant infection		
Yes	3	0
No	0	1
Unknown	0	1
Intravenous lines		
Yes	3	2
No	0	0
Immunocompromised^b^		
Yes	3	0
No	0	0
Unknown	0	2
Co-morbidities^c^		
Cancer	2	0
Transplant^d^	2	0
Diabetes	0	1
Hypertension	0	1

^a^ Wound swab (n = 1), cough swab (n = 1).

^b^ As reported by clinician.

^c^ Case(s) may have had more than one co-morbidity.

^d^ Renal transplant (n = 1), bone marrow transplant (n = 1).

The age of confirmed and probable cases ranged < 5 years to early sixties (median: 51 years). Three cases were male. Confirmed and probable cases were geographically dispersed. Three cases (two confirmed and one probable) were from outpatient settings, one confirmed case was from an inpatient setting and one probable case was from an unknown setting.

*Ralstonia pickettii* was retrieved from blood specimens for all confirmed cases and from swabs (one leg wound and one cough swab) for probable cases. All confirmed cases had clinically relevant infections requiring treatment (and have since recovered), at least one co-morbidity, were immunocompromised and had intravenous lines.

**Figure 2:**
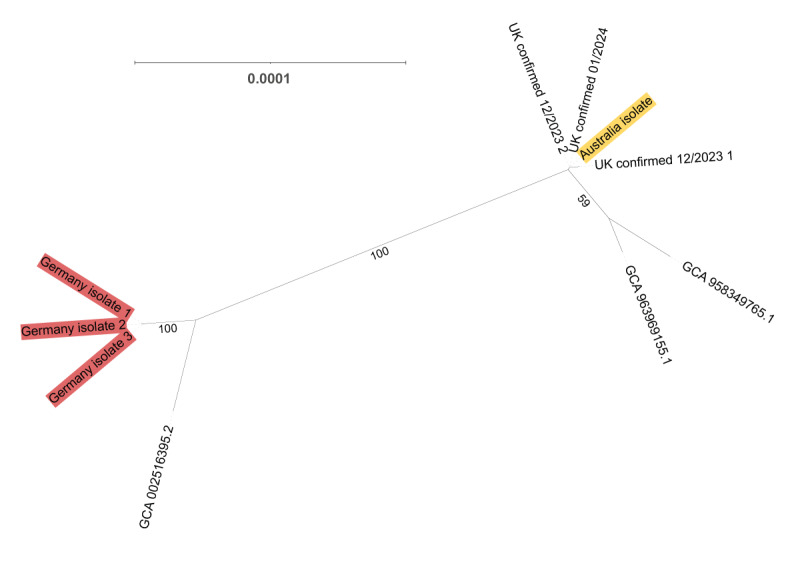
Phylogenetic reconstruction from whole genome sequences of *Ralstonia pickettii* isolates in the outbreak of the United Kingdom (n = 3) and comparison to isolates in Australia (n = 1) and Germany (n = 3)

GCA: GenBank genome assembly; UK: United Kingdom.

By comparative analysis of the WGS data, the three blood isolates from cases in the UK were genetically indistinguishable from the Australia strain with 0–4 SNP differences over 4,218,213 sites [4] (Figure 2). This suggested a point source outbreak with the UK and Australia isolates within the same cluster. Two additional isolates fitting the probable case definition were typed (distinct from probable cases described in Table) but found to have distinct PFGE profiles and > 30,000 SNPs (by SKA2 ‘dist’ tool to infer the number of nucleotide differences between genome sequences) from the prior cluster thus concluded to be unrelated to isolates of the confirmed cases. Isolates of the two probable cases detailed in Table were unavailable for typing. A further two isolates were typed and found not to be *Ralstonia* while a third was re-grown at the reporting laboratory and re-classified as a different organism. The UK isolates were also compared to isolates in Germany following a recent outbreak [3] and found to be considerably more divergent (hundreds of SNP differences), which was not suggestive of a common source between the UK and Germany isolates.

Maximum likelihood phylogeny inferred from a core genome alignment for the genomes similar to the Australia isolate. The Australia isolate is highlighted in yellow, and the Germany isolates in pink. The scale shown is in substitutions per site.

## Incident management, risk assessment and interventions

A multi-agency Incident Management Team was convened with representation from the UK devolved administrations, the UK regulator of medicines and medical devices, the health system and medicines supply specialists.

The benefits and risks of interventions including potential recall and impact on supply chain were considered. This risk assessment considered that products subject to suspension in Australia were widely distributed within the UK, including outside of clinical environments and available to the public online and use was expected to be poorly documented.

Actions implemented included issuing communications to the health system to assist case finding, requesting submission of isolates for typing, and informing stakeholders. The UK medicines regulator engaged with the manufacturer and suppliers. The UKHSA worked closely with partners to confirm a resilient supply of suitable alternative products. International partners were notified via EpiPulse [5] and via the International Health Regulation (IHR) National Focal Points (https://www.who.int/).

In March 2024, the manufacturer voluntarily recalled the associated products manufactured between April 2023 and November 2023 (noting production was paused thereafter) and issued a Field Safety Notice [6], which stated a leaking safety seal may have been responsible for water contamination during manufacture. The UKHSA communicated the product withdrawal to the health system and worked with the regulator to produce publicly accessible information with recommendations for healthcare providers and the public [7]. No new cases have arisen since the product was recalled.

## Discussion

*Ralstonia pickettii* (formerly *Pseudomonas pickettii)* is a low virulence non-fermentative Gram-negative bacterium found in soil and water [8]. It rarely causes disease in healthy individuals but can cause severe infection in people with immunocompromised status or cystic fibrosis. It has been implicated in numerous outbreaks of contaminated healthcare solutions including those classified as sterile [8-12]. The saline products associated with this outbreak were intended for inhalation, irrigation and eyewash only, however, *R. pickettii* bacteraemia occurred in immunocompromised people or people with co-morbidities.

In this outbreak, multi-agency investigation successfully identified cases linked to an outbreak crossing international borders and prompted a product recall to help prevent clinically relevant infection in people with underlying health conditions. There were, however, challenges, as *R. pickettii* is not a notifiable pathogen in the UK, may be dismissed as a contaminant or of unclear clinical significance and may be misidentified as *Burkholderia cepacia* complex [8], resulting in under-ascertainment. Furthermore, there was a broad range of potential populations and settings at risk of exposure because the associated saline solutions had different uses and were widely purchased for use in healthcare settings, non-clinical environments and by the public. Identifying products was challenged by inconsistent labelling, presentation as vials or in pre-assembled equipment packs, and these products lacked product codes to facilitate identification of recalled stock.

Our experience highlights the importance of prompt information sharing (including WGS and other typing information) and international collaboration to detect related incidents, and multi-agency and cross border working to investigate sources and implement necessary interventions. Based on our experience, public health institutions should adopt a low threshold to risk assess and investigate clusters of opportunistic pathogens like this and consider potential product contamination sources, especially when cases are geographically disseminated.

This outbreak is one of several recent, similar contamination incidents in the UK. Managing product contamination incidents is complex and affected products may be distributed globally. Though products labelled sterile are usually considered to have a high degree of quality assurance, our experience highlights potential risk of contamination from hydrophilic opportunistic pathogens.

## Conclusion

Though production of solutions associated with this outbreak was paused and products were recalled, products may have been widely distributed and if used, new cases may arise. International public health partners should be alert to potential cases and investigate new or recent isolates for genomic similarity with the UK-Australia strain. There is a need to prioritise prevention of such incidents and assure quality of healthcare products to protect the health of the public.
